# Cassava Starch-Based Thermo-Responsive Pb(II)-Imprinted Material: Preparation and Adsorption Performance on Pb(II)

**DOI:** 10.3390/polym14040828

**Published:** 2022-02-21

**Authors:** Meiyuan Lv, Yuhan Du, Tingting Zhang, Xueyu Du, Xueqiong Yin

**Affiliations:** Hainan Provincial Fine Chemical Engineering Research Center, Hainan University, Haikou 570228, China; meiyuan_lv@126.com (M.L.); duyuhan1109@126.com (Y.D.); tingting1210@126.com (T.Z.)

**Keywords:** thermo-responsive, Pb(II)-imprinted, cassava starch, adsorption, hydrogel

## Abstract

Heavy metal pollution is currently an increasing threat to the ecological environment, and the development of novel absorbents with remarkable adsorption performance and cost-effectiveness are highly desired. In this study, a cassava starch-based Pb(II)-imprinted thermo-responsive hydrogel (CPIT) had been prepared by using cassava starch as the bio-substrate, *N*-isopropyl acrylamide (NIPAM) as the thermo-responsive monomer, and Pb(II) as the template ions. Later, a variety of modern techniques including FTIR, DSC, SEM, and TGA were employed to comprehensively analyze the characteristic functional groups, thermo-responsibility, morphology, and thermal stability of CPIT. The obtained material exhibited superior performance in adsorption of Pb(II) and its maximum adsorption capacity was high—up to 114.6 mg/g under optimized conditions. Notably, the subsequent desorption (regeneration) process was fairly convenient by simply rinsing with cold deionized water and the highest desorption efficiency could be achieved as 93.8%. More importantly, the adsorption capacity of regenerated CPIT still maintained 88.2% of the value of starting material even after 10 recyclings. In addition, the excellence of CPIT in selective adsorption of Pb(II) should also be highlighted as its superior adsorption ability (97.9 mg/g) over the other seven interfering metal ions.

## 1. Introduction

Accompanied by the rapid development of industrial society, heavy metal pollution has become an increasingly severe threat to ecological environment due to its extremely high toxicity, carcinogenicity, and biological enrichment tendency. As a major type of wide spread pollutants on the earth, lead ions (Pb(II)) stem from the industrial effluents from metal smelting, mining, painting, battery, and leather industries [1]. To effectively diminish the consistency of Pb(II) in aquatic environment, different treatments have been therefore developed, including chemical precipitation, electrochemical process, ion exchanging, membrane separation, adsorption method, etc. [2,3]. It is noteworthy that the adsorption method is considered a highly promising approach featured with simplified operation, flexible design, and convenience for recycle [4,5]. To date, many types of adsorbents have been attempted for adsorption of Pb(II), e.g., carbon nanotubes [6], ferro-manganese binary oxide [7], and kaolinite [8]. However, the employment of these materials is commonly restricted by specific adverse aspects such as complicated production processes, high costs, *etc.* Therefore, it is of great significance to develop a more competitive adsorbent for Pb(II) on the premise of superior adsorption property and low production cost. 

Ion-imprinted technology (IIT), developed from molecular imprinting technology (MIT), is a specialized technique for synthesis of ion-imprinted polymer (IIP) that could be employed as an efficient adsorbent with outstanding capacity of selective recognition and adsorption of heavy metal ions [9]. During the preparation process of IIP, the target template ions firstly coordinate with functional monomers to form complexes that further proceed to be polymerized with the presence of a cross-linking agent. After removal of template ions, the specific binding sites would be formed, highly matching the template ions with unique size, charge, coordination number, geometrical shape, and functional groups [10,11]. To date, relevant efforts have been pursued to promote the applications of IIP for selective recognition and recombination of target heavy metal ions from their aqueous solution. For instance, the imprinted material fabricated by bacterial cellulose (matrix) and N-isopropyl acrylamide (temperature-sensitive monomer) exhibited a maximum adsorption capacity of Cu^2+^ as 140.9 mg/g, if pre-embedding Cu^2+^ as template ions. Moreover, its high selectivity was also reflected by the fact that it could selectively adsorb Cu^2+^ to a high level of 81.9 mg/g from a solution coexisted with seven other interfering ions (0.5–9.0 mg/g) [12]. Recently, a novel Pb(II)-imprinted polymer (MnFe_2_O_4_@SiO_2_/GO-IIP) applying magnetic and lamellar graphene oxide was successfully synthesized by surface ion-imprinted technique. Its maximum adsorption capacity of Pb(II) in aqueous solution was achieved as 58.8 mg/g within 30 min and more remarkably the resultant adsorbent could be readily separated by a permanent magnet. Furthermore, its high stability and reusability had also been verified from the results of regeneration experiments [13].

Recently, green polymer-based hydrogels have drawn extenstive attention for their versatile potentials in the applications of biomedicine and environmental remediation [14,15]. They could also be served as base materials for IIP, whose chemical structure varies following the changes of external-environment factors (e.g., temperature, pH, illumination, electromagnetic effect, etc.) [16]. Take the currently most investigated one, namely thermo-responsive hydrogel as an example, its interior and exterior structure would be spontaneously varied when the ambient temperature changes. Chen et al. reported successfully synthesizing a thermo-magneto-responsive poly (*N*-isopropyl acrylamide)-magnetite composite hydrogel and its adsorption of Cr^3+^ occurred primarily in the temperature range of 25–50 °C. When the temperature dropped below 25 °C, desorption of Cr^3+^ turned out to be the prevailing process. The maximum adsorption efficiency was achieved as 60.9% when the initial concentration of Cr^3+^ was 20 mg·L^−1^ [17].

In the current study, we designed and prepared a cassava starch based Pb(II)-imprinted thermo-responsive material (CPIT) by the combination of IIT and thermo-responsive technology via a gradient heating polymerization methodology. *N*-isopropylacrylamide (NIPAM) was applied as the thermo-responsive monomer, which was also reported to be an important monomer material for preparation of graft copolymers [18,19]. In addition, Pb(II) and cassava starch were adopted as target template ions and substrate, respectively. Then, CPIT was obtained after thorough removal of template ions Pb(II) with deionized water below its phase transition temperature. Compared with the existing heavy metal adsorbents, this type of intelligent material is aiming to effectively and selectively adsorb Pb(II) from wastewater by simple temperature controlling. The eco-friendly regeneration of CPIT realized by cold water rinsing is attributed to its unique thermo-responsibility. When the temperature is lower than the phase transition temperature of CPIT, the material swells and releases the adsorbed Pb(II). In turn, if the treatment temperature is higher than the phase transition temperature of CPIT, the material shrinks and regenerates the recognition sites for Pb(II). In addition, the structural information and property of CPIT had been revealed in terms of phase transition behavior, morphology, characteristic functional groups, and thermal stability. Its adsorption and desorption performance of Pb(II) as well as the corresponding adsorption mechanism were also studied prior to the investigation of selective adsorption capacity on Pb(II) with existence of other interfering metal ions. Moreover, the regeneration experiments of CPIT were carried out to evaluate its potentials for large scale applications. It is expected that the current work might serve as a theoretical and practical guide for preparation of other types of adsorbents aiming for different scenarios by applying specific template ions or substrates, which would definitely promote the industrialized applications of ion-imprinted polymers.

## 2. Materials and Methods

### 2.1. Materials

Cassava starch (CS) was purchased from Hainan Herixiang Food Co., Ltd. (Haikou China). *N*-isopropyl acrylamide (NIPAM), ammonium persulfate (AP), and *N*, *N*′-methylenebisacrylamide (BIS) were supplied from Macklin Biochemical Co., Ltd. (Shanghai, China). Potassium chloride, sodium chloride, lead nitrate, and nitric acid were purchased from Guangzhou Chemical Reagent Co., Ltd. (Guangzhou, China).

### 2.2. Characterization

Thermal behavior of CPIT was determined by a Q100 differential scanning calorimeter (Setara Instruments, Lyon, France) under N_2_ atmosphere in the range from 10 °C to 80 °C. Both the heating and cooling rates were set as 10 °C/min. FTIR spectra of CS, NIPAM, and CPIT were analyzed with a TENSOR 27 FTIR spectrometer (Bruker Corporation, Karlsruhe, Germany) by KBr pellet method. All spectra were recorded in the range from 4000 cm^−1^ to 500 cm^−1^. Thermal stability of poly (*N*-isopropyl acrylamide) (PNIPAM) and CPIT was evaluated by using a SDT Q600 thermogravimetric analyzer (TA Instruments, New Castle, DE, United States) under a nitrogen atmosphere and test temperature was programed from 10 to 880 °C with a heating rate as 10 °C/min. The surface morphology of lyophilized CPIT was observed by using an S-3000N scanning electron microscope (Hitachi, Tokyo, Japan) with an accelerating voltage of 10 kV.

### 2.3. Preparation of CPIT

**Figure 1:**
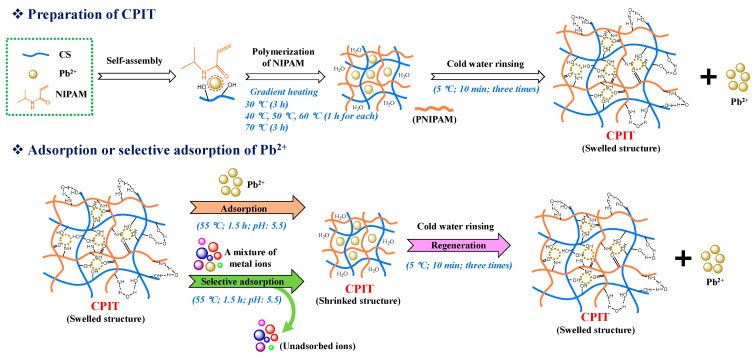
Preparation and functional mechanism diagram of CPIT.

CPIT was polymerized by a gradient heating procedure [20]. Its detailed preparation process and the mechanisms of adsorption and desorption of Pb(II) are presented in (Figure 1). Briefly, 1.00 g CS, 0.15 g BIS, 2.11 g AP, a certain amount of NIPAM and Pb(II) solution (2 mg/mL), and 100 mL deionized water were successively added to a three-necked round-bottom flask that was submerged into a water bath with magnetic stirring. The reaction was initiated at 30 °C and lasted for 3 h. After that, the temperature was controlled at 40 °C, 50 °C, and 60 °C for 1 h each, and then maintained at 70 °C for 3 h. CPIT was obtained after rinsing the resultant product thoroughly with deionized water (5 °C) under vacuum filtration to remove the pre-imprinted Pb(II) and then stored at 10 °C for future use. It was determined that around 99% of the trapped template ions had been rinsed off according to the purification step mentioned above.

### 2.4. Adsorption Performance and Mechanism of CPIT

The effects of adsorption temperature, adsorption time, pH value of initial solution, and initial concentration of Pb(II) were investigated by single factor experiments. During each run of adsorption, a given amount of wet CPIT (equivalent to 0.1 g dry weight) together with 20 mL lead nitrate solution (initial concentration of Pb(II): 0.05–2.0 mg/mL) was introduced into a 50 mL beaker. After adsorption by CPIT under pre-set conditions, the Pb(II) concentration of the resultant solution was determined by a TAS-990SUPER AFG flame atomic adsorption spectrophotometer (Beijing Purkinje General, Beijing, China). The actual (Q_t_) and equilibrium (Q_e_) adsorption capacity were calculated by Equations (S1) and (S2) (Supplementary Materials).

The pseudo-first-order and pseudo-second-order models were used to simulate the adsorption kinetics of CPIT following Equations (S3) and (S4) (Supplementary Materials). Furthermore, the isothermal adsorption data was fitted by Langmuir and Freundlich models, expressed by Equations (S5) and (S6) (Supplementary Materials).

### 2.5. Desorption and Regeneration of CPIT

Pretreat a certain amount of CPIT in 1.7 mg/mL Pb(II) solution for 1.5 h to realize saturated adsorption. Later, the treated sample was rinsed with deionized water for three times under vacuum microfiltration to release Pb(II). Selection of suitable desorption conditions in terms of desorption temperature (5–30 °C), desorption time (5–30 min), and rinsing times (1–5) were also studied by single factor experiments and the desorption efficiency (D%) was determined according to Equation (S7) (Supplementary Materials). The regeneration of CPIT was carried out by applying all the suitable desorption conditions screened above. The process of adsorption-desorption was repeated up to 10 times for evaluating the adsorption capacity and desorption efficiency of the ultimate product.

### 2.6. Selective Adsorption of CPIT

A fixed amount of CPIT was submerged in a mixed solution containing Pb^2+^, Cd^2+^, Mg^2+^, Ca^2+^, Cu^2+^, Fe^3+^, Na^+^, and K^+^. The initial concentration of each metal ion was prepared as 1.7 mg/mL. After adsorption for 1.5 h at 55 °C, the resultant sample was treated according to the similar procedure as described in Section 2.4.

## 3. Results and Discussion

### 3.1. Characterization of CPIT

**Figure 2:**
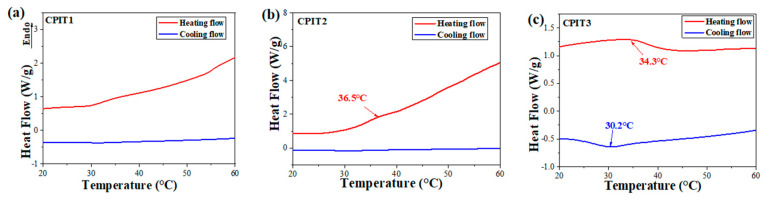
Effect of molar ratio of AGU to NIPAM on thermo-responsibility of CPIT. (**a**) n_AGU:_n_NIPAM_ = 1:3; (**b**) n_AGU:_n_NIPAM_ = 1:4; (**c**) n_AGU:_n_NIPAM_ = 1:5.

The thermo-responsibility of different CPIT samples was reflected from their individual DSC thermograms. To better optimize the amounts of CS and NIPAM for preparation of CPIT, different molar ratios of anhydroglucose units (AGU) in CS to NIPAM were adopted. The mole number of AGU in CS (denoted as n_AGU_) was obtained by dividing the mass of CS by the moalr mass of AGU (162 g/mol). As demonstrated in (Figure 2a,b), there are no obvious endothermic or exothermic peaks observed for CPIT1 (n_AGU_:n_NIPAM_ = 1:3) and CPIT2 (n_AGU_:n_NIPAM_ = 1:4) during corresponding heating or cooling process. This might be due to the fact that a relatively low amount of NIPAM could not contribute to a desired degree of polymerization, which is the prerequisite of achieving reversible thermo-responsibility. It is necessary to note that, when the molar ratio of NIPAM to AGU further increases up to 5, as for CPIT3 (n_AGU_:n_NIPAM_ = 1:5), an evident endothermic peak appears at 34.3 °C during heating scan from 20 °C to 60 °C. Besides, an exothermic peak is also emerged around 30.2 °C from its cooling scan. The above results indicated that a reversible thermo-responsive structure had been formed in CPIT by applying a proper molar ratio between AGU and NIPAM as 1:5.

**Figure 3:**
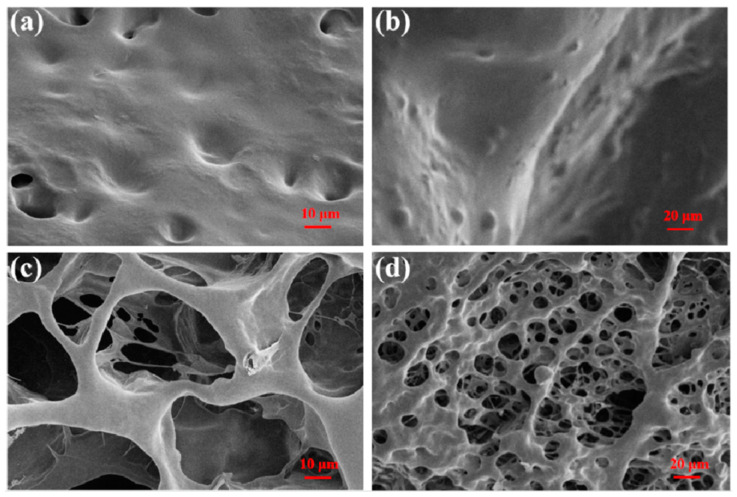
SEM images of CPIT samples after different treatments. (**a**) After adsorption of Pb(II) at 70 °C with high magnification; (**b**) After adsorption of Pb(II) at 70 °C with low magnification; (**c**) After desorption of Pb(II) at 10 °C with high magnification; (**d**) After desorption of Pb(II) at 10 °C with low magnification.

The surface morphology of CPIT samples after adsorption and desorption of Pb(II) is presented in (Figure 3). As shown in (Figure 3a,b), the surface of CPIT loaded with Pb(II) is full of concave–convex structures, but with limited small voids. On the contrary, a large number of holes evenly distributed in the sponge-like sample is observed in (Figure 3c,d). This obvious difference in surface morphology accounts for the fact that the thermo-responsibility of CPIT had been realized. In practical application, the voids existing in CPIT shrink, recognize, and adsorb Pb(II) when the temperature increases. As a consequence, the pores present in CPIT would be gradually filled up and disappear. When the material cools down, resulting in a more expanded and loosened structure, which facilitates desorption of Pb(II) and reappearance of the pores. In general, the purpose of adsorption or desorption could be readily manipulated by adjusting the ambient temperature higher or lower than its corresponding PTT. 

**Figure 4:**
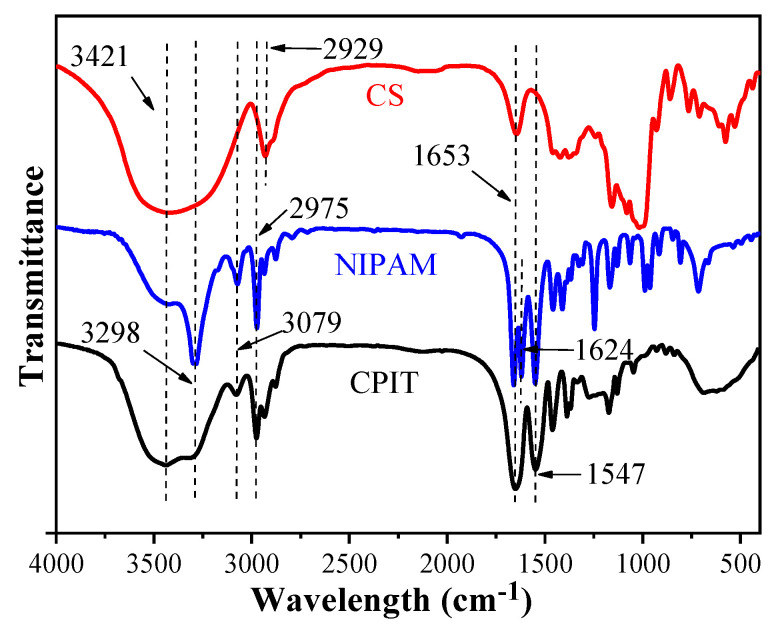
FTIR spectra of CS, NIPAM, and CPIT.

The FTIR spectra of CS, NIPAM, and CPIT are illustrated in (Figure 4). For CS, the broad peak around 3421 cm^−1^ is the O–H stretching vibration of hydroxyl groups. The adsorption peaks at 2929 cm^−1^ and 1653 cm^−1^ are attributed to the C–H and C=O stretching vibrations from glucosl units in CS, respectively [21,22]. Regarding NIPAM, a characteristic absorption peak at 3298 cm^−1^ refers to the N–H stretching vibration of amino groups and the absorption of C–H stretching vibration at 2975 cm^−1^ originates from the methyl groups. The absorption peaks at 1653 cm^−1^ and 1624 cm^−1^ are related to C=O stretching vibration of amide I band and C=C stretching vibration, respectively. Moreover, the absorption peaks of N–H bending vibration and C–N stretching vibration from amide groups are overlapped around 1547 cm^−1^. 3079 cm^−1^ is the overtone band of N-H bending vibration [23]. In the spectrum of CPIT, the adsorption bands at 3421 cm^−1^, 3298 cm^−1^, 3079 cm^−1^, 1653 cm^−1^, and 1547 cm^−1^ are corresponding to O–H stretching vibration, N–H stretching vibration, N–H overtone, C=O stretching vibration, and N–H bending vibration, respectively. It is worth noting that the characteristic absorption peaks stemming from CS and NIPAM are also observed in the spectrum of CPIT, e.g., the C=O stretching vibration (1653 cm^−1^) and the N–H bending vibration (1547 cm^−1^).

**Figure 5:**
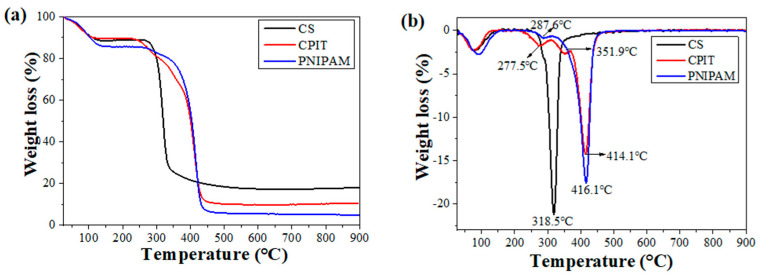
TG (**a**) and DTG (**b**) curves of CS, CPIT, and PNIPAM.

The thermal stabilities of CS, CPIT, and PNIPAM are shown in (Figure 5). In general, the weight loss occurring below 120 °C is corresponding to the removal of free and bound water for all test specimens. In addition to that, a primary weight loss stage observed for CS is ranging from 250 °C to 430 °C and the temperature for maximum weight loss rate (T_DTGmax_) is 319 °C (Figure 5b). For PNIPAM, its thermal decomposition process could be described as two periods: (i) 202–315 °C with T_DTGmax_ as 288 °C; (ii) 315–469 °C with T_DTGmax_ as 416 °C (Figure 5b). These two weight loss stages are resulted from the degradation of amide groups and backbones of PNIPAM, respectively [24,25]. Compared with CS and PNIPAM, the DTG curve of CPIT is featured as three weight loss steps (First: 202–312 °C, T_DTGmax_: 278 °C; Second: 312–369 °C, T_DTGmax_: 352 °C; Third: 369–469 °C, T_DTGmax_: 414 °C), which is obviously different from the outcome of simply overlapping the curves of CS and PNIPAM. In other words, during the preparation process of CPIT, the NIPAM monomer did get grafted onto the molecular chains of CS and then further self-polymerized to prolong the chains of PNIPAM, instead of physical blending of CS and PNIPAM. Therefore, all the weight-loss characteristics of PNIPAM has been incorporated in the DTG curve of CPIT. Moreover, an additional weight loss interval observed for CPIT ranging from 312–369 °C (the second step), to a large extent, owing to the presence of segments crosslinked between CS and PNIPAM.

### 3.2. Adsorption Performance of CPIT on Pb(II)

#### 3.2.1. Effect of Temperature on Pb(II) Adsorption by CPIT

**Figure 6:**
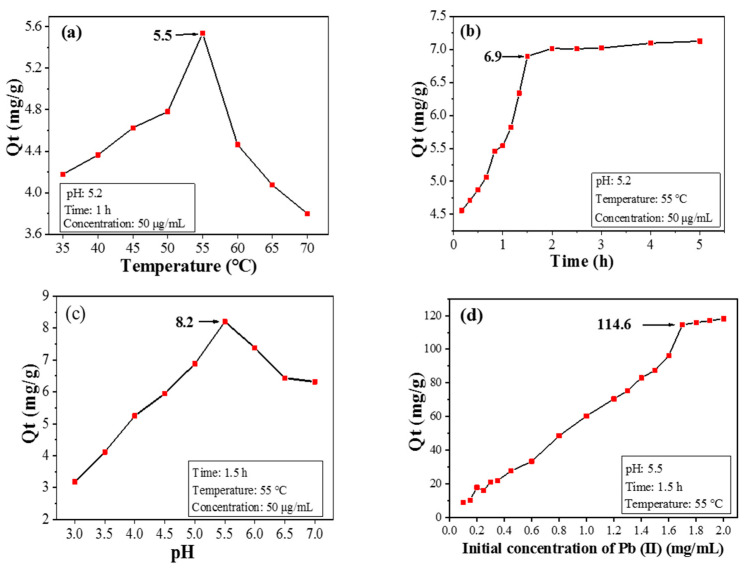
Effects of temperature (**a**), time (**b**), pH value (**c**), and initial Pb(II) concentration (**d**) on adsorption performance.

The adsorption capacity of CPIT towards Pb(II) increases first and then drops within the temperature range from 35 °C to 70 °C, during which the maximum value of Q_t_ is acquired as 5.5 mg/g when 55 °C is applied (Figure 6a). This phenomenon is reasonable since low temperature (e.g., 35 °C) favors CPIT in a more swollen and loosened state. Consequently, the pores with enlarged size are not conducive to capture Pb(II). When the temperature gradually rises, the hydrogen bonds between the material and water molecules would be impaired, causing the CPIT to shrink. The persistent reduction in pore size response from 35 °C to 55 °C is beneficial for the adsorption and retention of Pb(II). After achieving the maximum Q_t_ value at 55 °C, however, the adsorption capacity of CPIT declines sharply, ascribed to the over-shrinked pore size which adversely affects the embedding of Pb(II). Moreover, the tendency of desorption under high temperature should be another reason. 

#### 3.2.2. Effect of Adsorption Time on Pb(II) Adsorption by CPIT

A rapid growth of adsorption capacity is observed when the adsorption time is prolonged up to 1.5 h, and the maximum Q_t_ value is achieved as 6.9 mg/g (Figure 6b). During the early stage of adsorption, there are a large number of effective recognition sites available both on the surface and inside the structure of CPIT. This fact is in line with the potent increasing trend of adsorption capacity from 0 to 1.5 h. Later, as the amount of adsorbed Pb(II) continues to accumulate, majority of the recognition sites would be occupied, leaving less amount of hollow recognition sites. Moreover, the adsorbed Pb(II) from the recognition sites would also repel the dissociated ones in solution via electrostatic repulsion, so the adsorption rate would gradually decrease until attaining equilibrium. Based on the discussion above, a suitable adsorption time could be selected as 1.5 h. 

#### 3.2.3. Effect of pH Value on Pb(II) Adsorption by CPIT

The adsorption capacity of CPIT on Pb(II) also varies according to different initial pH value of the Pb(II)-containing solution. When the pH value is only 3, the surface of CPIT would be extensively protonated, resulting in strong charge repulsion towards positively charged Pb(II) [26]. In this occasion, less amount of Pb(II) would overcome the charge repulsion force to be adsorbed by CPIT, and thus the adsorption capacity is fairly low as 3.2 mg/g (Figure 6c). As the pH value climbs up, the electrostatic attraction instead of charge repulsion takes a major role in propelling dissociated Pb(II) towards CPIT [27]. Additionally, controlled polymerization of CPIT with amide groups capable for metal-ion chelation takes part in the capture of Pb(II) [28]. The maximum adsorption capacity is acquired as 8.2 mg/g, when pH value reaches 5.5 (Figure 6c). It is necessary to point out that further elevation of pH value unexpectedly decreases the adsorption capacity. The reason is largely due to the partial hydrolysis of Pb(II) into Pb(OH)_2_ that possesses fairly low solubility in a weak acid environment and therefore adversely impacts the adsorption capacity of CPIT [29]. 

#### 3.2.4. Effect of Initial Pb(II) Concentration on Pb(II) Adsorption by CPIT

Generally speaking, higher concentration of initial Pb(II) aqueous solution benefits the achievement of higher adsorption capacity of Pb(II). When the initial Pb(II) concentration reaches 1.7 mg/mL, the adsorption capacity climbs to a plateau (114.6 mg/g), much higher than the values achieved by applying a silica-based molecularly imprinted mesoporous polymer (23.8 mg/g) [30] and a chitosan-based composite membrane (76.6 mg/g) [31]. However, there is no evident increase of Q_e_ value for CPIT if the initial Ph(II) concentration exceeds 1.7 mg/mL.

### 3.3. Adsorption Mechanism of CPIT

**Figure 7:**
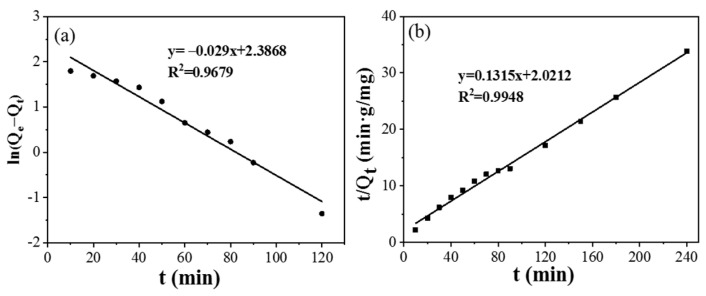
Adsorption kinetics of CPIT on adsorption of Pb(II) by using pseudo–first–order model (**a**) and pseudo–second–order model (**b**).

The adsorption kinetics of CPIT was studied by pseudo-first-order and pseudo-second-order models. Relevant simulation parameters and results are summarized in (Figure 7) and (Table 1). In comparison to the pseudo-first-order model (R^2^ = 0.9502), the pseudo-second-order model (R^2^ = 0.9948) is more fitting to the adsorption process of CPIT, which indicates that the adsorption process is primarily driven by the chemical adsorption between the imprinted cavities from CPIT and Pb(II) via coordination, hydrogen bonds, and electrostatic interaction [32]. The correlation between the equilibrium concentration of Pb(II) in solution and the maximum adsorption capacity at equilibrium are fitted by the Langmuir and Freundlich adsorption isotherm models, respectively (Table 2). As shown in Table 2, the adsorption of Pb(II) by CPIT is better fitted by Freundlich model, reflected by its higher fitting degree (R_F_^2^ = 0.9839). This indicates that the removal of Pb(II) is driven by a heterogeneous multi-molecular layer adsorption [33]. 

### 3.4. Desorption Performance of CPIT

**Figure 8:**
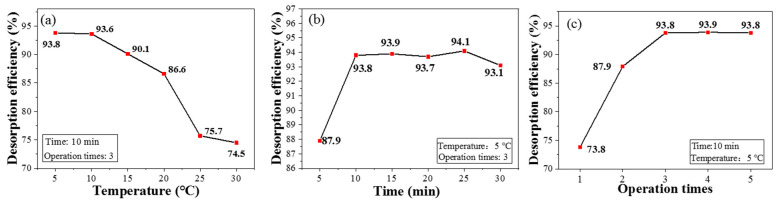
Desorption rates of CPIT at different temperature (**a**), immersion time (**b**), and operation times (**c**).

Compared with currently environment-unfriendly desorption approaches, e.g., using nitric acid as an eluting agent [34], CPIT exhibits outstanding superiority to realize desorption via cold deionized water rinsing. As shown in Figure 8a, the desorption efficiency of CPIT decreases with the elevation of temperature and the maximum value is achieved as 93.8% when the temperature is controlled at 5 °C. As mentioned before, low temperature enlarges the pore size of active recognition sites and meanwhile weakens the interactions between Pb(II) ions and CPIT molecular chains, which eventually promotes the desorption of Pb(II). As demonstrated in Figure 8b, the desorption efficiency increases rapidly from 5 to 10 min of immersion time, and gradually closes to the equilibrium when the time exceeds 10 min. Besides, the effect of rinsing times on desorption is also plotted in Figure 8c. As expected, the desorption efficiency of CPIT does elevate with the increment of rinsing times and finally reaches the plateau (around 93.8%) after being rinsed three times.

Since the adsorption process of Pb(II) exhibits a better fitting to heterogeneous multi-molecular layer adsorption, the Pb(II) on the surface layer is preferentially desorbed during rinsing. Later, the Pb(II) embedded in the inner layer would be gradually desorbed due to multiple factors—e.g., the expansion of polymer chains, the destruction of the interaction between ions and polymer functional groups, and the enlarging of pores [35]. Therefore, the suitable desorption conditions could be summarized as 5 °C of desorption temperature, 10 min of immersion, and three rinsings.

### 3.5. Selective Adsorption Capacity of CPIT

**Figure 9:**
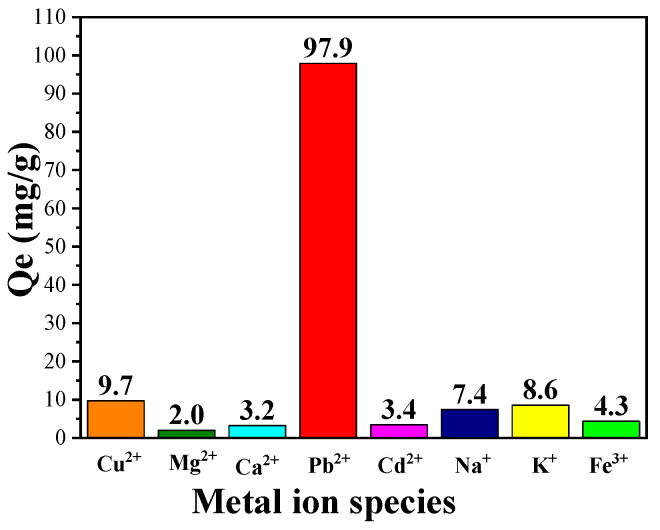
Selective adsorption capacity of CPIT on Pb(II) from a mixed metal-ion solution containing eight metal ions (namely Cu^2+^, Mg^2+^, Ca^2+^, Pb^2+^, Cd^2+^, Na^+^, K^+^, and Fe^3+^).

In addition to the remarkable adsorption capacity of CPIT on single solute solution of Pb(II), its ability of selective adsorption of Pb(II) from solution containing multiple ions was also investigated. As demonstrated in (Figure 9), CPIT exhibits an outstanding capacity of selective adsorption of Pb(II) up to 97.9 mg/g from a solution containing eight metal ions in total (namely, Cu^2+^, Mg^2+^, Ca^2+^, Pb^2+^, Cd^2+^, Na^+^, K^+^, and Fe^3+^), while the adsorption efficiency of other seven ions is in the range from 2.0 to 9.7 mg/g. This result is rational, for all the active sites were previously imprinted by Pb(II), and all these cavities may not be suitable for matching the metal ions with different diameters and/or charge numbers. However, the presence of other metal ions would inevitably interfere the adsorption of Pb(II) in a minor extent. 

### 3.6. Regeneration of CPIT

**Figure 10:**
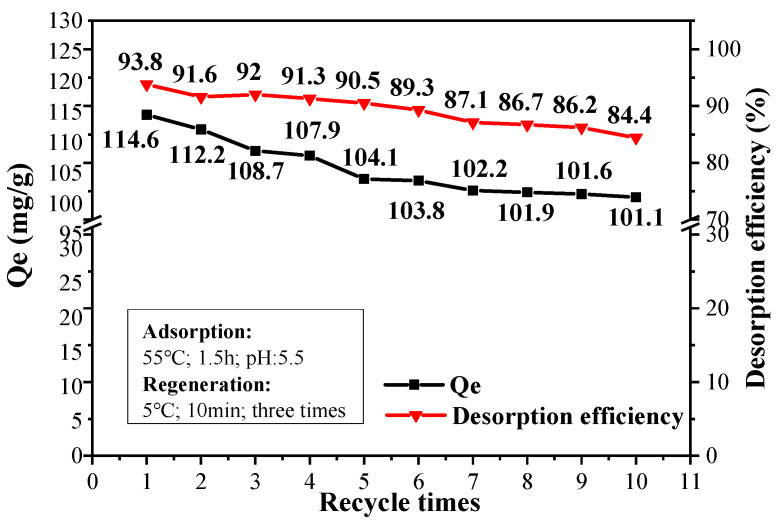
Adsorption and desorption efficiency of CPIT for up to 10 recyclings.

A slight reduction trend is observed for both Q_e_ value and desorption efficiency of CPIT with increased recycle times (Figure 10). After 10 recyclings, the adsorption capacity still remained at 101.1 mg/g compared with 114.6 mg/g of the pristine material. The desorption efficiency, on the other hand, drops from 93.8% to 84.4%. This is mainly due to a trace amount of Pb(II) remaining in the eluted material after each time of desorption, which brings about the accumulated reduction of effective adsorption sites in CPIT. In general, the maximum adsorption capacity of CPIT is still lower than that of magnetic activated carbon [36] and some other recently reported adsorbent [37], but its greatest advantages could be highlighted as an eco-friendly generation process simply by cold water (5 °C) rinsing instead of acid/base treatment. Moreover, its adsorption and desorption efficiency could still remain 88.2% and 90.1% of its starting values after 10 recyclings, more remarkable than those of some other high-performance adsorbents recently documented [37,38].

## 4. Conclusions

Via a gradient heating procedure, a cassava-based Pb(II)-imprinted thermo-responsive hydrogel (CPIT) had been successfully prepared by applying cassava starch as the substrate, *N*-isopropyl acrylamide (NIPAM) as the thermo-responsive monomer, and Pb(II) as the template ions. The reversible thermo-responsibility of the obtained material had been revealed in the range from 30.2 °C to 34.3 °C and the maximum adsorption capacity and desorption efficiency of Pb(II) were determined as 114.6 mg/g and 93.8%, respectively. Moreover, CPIT also exhibits superior capacity in selective adsorption and regeneration. Its adsorption capacity of Pb(II) still remains 88.2% of the value of starting material after 10 recyclings. Therefore, this novel type of intelligent bio-based adsorbent has tremendous application potential in the field of heavy metal sewage treatment. 

## Figures and Tables

**Table 1:** Fitting results on Pb(II) adsorption by using pseudo-first-order and pseudo-second-order models.

Samples	Pseudo-First-Order Model			Second-Order Dynamic		
	k_1_	R^2^	Q_e_	k_2_	R^2^	Q_e_
CPIT	0.0005	0.9502	7.0554	0.0086	0.9948	7.6046

**Table 2:** Parameters of Langmuir and Freundlich adsorption isotherms of Pb(II) adsorption.

Adsorption Isotherms of Pb(II)						
Samples	Langmuir Model			Freundlich Model		
	K_L_ (L/mg)	Q_m_ (mg/g)	R_L_^2^	K_F_ (L/mg)	n	R_F_^2^
CPIT	0.0008	189.39	0.9250	0.5006	1.3298	0.9839

## Data Availability

Not applicable.

## Supplementary Materials

The following supporting information can be downloaded at: https://www.mdpi.com/article/10.3390/polym14040828/s1.
