# Supplementary material for: Cassava Starch-Based Thermo-Responsive Pb(II)-Imprinted Material: Preparation and Adsorption Performance on Pb(II)
Source: Polymers (Basel). 2022 Feb 21;14(4):828. doi: 10.3390/polym14040828 (PMC8963116; doi:10.3390/polym14040828)
Supplement: Supplementary file 1 [file polymers-14-00828-s001.zip › polymers-1589116-supplementary.pdf]

Supplementary Material

Cassava Starch-Based Thermo-Responsive Pb(II)-Imprinted Material: Preparation and Adsorption Performance on Pb(II)

Meiyuan Lv, Yuhan Du, Tingting Zhang, Xueyu Du*, Xueqiong Yin*

2.4. Adsorption performance and mechanism of CPIT

$$Q_t = \frac{(c_0 - c_t) \times V}{W} \qquad (S1)$$

$$Q_e = \frac{(c_0 - c_e) \times V}{W} \qquad (S2)$$

Where, c_0 is the initial Pb(II) concentration of the solution prior to adsorption, mg/mL; c_t is the Pb(II) concentration of the solution after adsorption, mg/mL; c_e is the equilibrium Pb(II) concentration of the solution after adsorption, mg/mL; V is the total volume of the treated solution, mL; W is the dry weight of CPIT, g.

$$\ln(Q_e - Q_t) = \ln Q_e - k_1 t \qquad (S3)$$

$$\frac{t}{Q_t} = \frac{1}{k_2 \cdot {Q_e}^2} + \frac{t}{Q_e} \qquad (S4)$$

Where, Q_e is the equilibrium adsorption capacity, mg/g; Q_t is the adsorption capacity at time t, mg/g; k_1 is the rate constant of the first-order kinetics, min^{-1}; k_2 is the rate constant of the second-order kinetics, g/(mg·min).

$$\frac{1}{Q_e} = \frac{1}{Q_m} + \frac{1}{K_1 \cdot C_e \cdot Q_m} \qquad (S5)$$

$$\ln Q_e = lnK_f + \frac{1}{n} lnC_e \qquad (S6)$$

Where, Q_m is the theoretical maximum value of adsorption capacity, mg/g; C_e is the equilibrium value of Pb(II) concentration, g/L; K_l is the Langmuir adsorption equilibrium constant, L/mg; K_f and n are two constants for Freundlich isotherm model at a specific temperature.

2.5. Desorption and regeneration of CPIT

$$D\% = \frac{C_d \times V}{Q_e \times W} \times 100\% \qquad (S7)$$

Where, C_d is the Pb(II) concentration of the resultant solution after desorption, mg/mL; V is the total volume of the resultant solution, mL; Q_e is the equilibrium adsorption capacity of CPIT, mg/g; W is the dry weight of CPIT, g.
